# Class I PI3K regulatory subunits control differentiation of dendritic cell subsets and regulate Flt3L mediated signal transduction

**DOI:** 10.1038/s41598-022-16548-x

**Published:** 2022-07-19

**Authors:** Keyur Thummar, Chozha Vendan Rathinam

**Affiliations:** 1 Department of Genetics and Development, Columbia University Medical Center, New York, NY 10032 USA; 2 Institute of Human Virology, University of Maryland School of Medicine, 725 West Lombard Street, Baltimore, MD 21201 USA; 3 Center for Stem Cell and Regenerative Medicine, University of Maryland School of Medicine, 725 West Lombard Street, Baltimore, MD 21201 USA

**Keywords:** Immunology, Haematopoiesis, Innate immune cells

## Abstract

Dendritic cells (DCs) play pivotal roles in initiating and shaping both innate and adaptive immune responses. The spatiotemporal expression of transcription factor networks and activation of specific signal transduction pathways determine the specification, distribution and differentiation of DC subsets. Even though pioneering studies have established indispensable roles for specific catalytic subunits (p110δ and p110γ) in immune cells, functions of the regulatory subunits, particularly of Class I PI3K, within the hematopoietic system remain incompletely understood. In the study presented here, we deleted the key regulatory subunits—p85α and p85β of the Class I_A_ PI3K in hematopoietic cells and studied its impact on DC differentiation. Our studies identify that a deficiency of p85 causes increased differentiation of conventional DC (cDC) 2 and plasmacytoid DC (pDC) subsets in the spleen. On the other hand, DC numbers in the bone marrow (BM), thymus and lymph nodes were decreased in p85 mutant mice. Analysis of DC-specific progenitors and precursors indicated increased numbers in the BM and spleen of p85 deficient mice. In-vitro differentiation studies demonstrated augmented DC-differentiation capacities of p85 deficient BM cells in the presence of GM-CSF and Flt3L. BM chimera studies established that p85 deficiency affects DC development through cell intrinsic mechanisms. Molecular studies revealed increased proliferation of DCs and common DC progenitors (CDPs) in the absence of p85 and altered signal transduction pathways in p85 mutant DC subsets in response to Flt3L. In essence, data presented here, for the first time, unequivocally establish that the P85α subunit of class I_A_ PI3Ks has an indispensable role in the development and maintenance of DCs.

## Introduction

Dendritic cells (DCs) play pivotal roles in initiating and shaping both innate and adaptive immune responses^1,2^. In particular, DCs are essential for the antigen presentation to effector cells and initiation of protective T cell responses and, therefore, responsible for a frontline defense against immune insults^3–5^. DCs are broadly divided into 3 major categories based on specific immunophenotype; 1. CD11c^+^CD8^+^ Classical or conventional (c) DC1 (cDC1/ lymphoid DCs); 2. CD11c^+^CD11b^+^ cDC2 (myeloid DCs); and 3. CD11c^+^PDCA1^+^ plasmacytoid DCs (pDCs)^4,6,7^. The spatiotemporal expression of transcription factor networks and activation of specific signal transduction pathways determine the specification, distribution and differentiation of different DC subsets in lymphoid and non-lymphoid tissues. Indeed, deregulated expression of transcription factors and defective signaling pathways lead to impaired DC differentiation and functions, and ultimately result in severe autoimmune deficiencies and enhanced susceptibility to viral, bacterial and fungal infections in mice and humans^8–10^. Of note, DCs have long been considered ideal candidates for targeted vaccine approaches, but this promise remains largely unfulfilled^3^, due to lack of a comprehensive knowledge on the mechanisms that control their development and functions. Understanding the molecular control of DCs is likely to have important implications in the treatment of immunological disorders in humans.

Phosphatidylinositol-3-kinase (PI3K) pathways play fundamental roles in various cellular processes, including T and B cell receptor signaling, activation of G protein-coupled receptors, Toll- like receptors (TLR) signaling and cytokine production^11,12^. PI3Ks are a subfamily of lipid kinases that are responsible for adding a phosphate group to the 3-position of the inositol ring of three species of phosphoinositol (PI) lipid substrates; PI, PI-4-phosphate PI(4)P, and PI-4,5-bisphosphate PI(4,5)P_2_^13–15^. PI3K family of enzymes is broadly classified into class I (I_A_ and I_B_), class II and class III. Class I_A_ consists of protein subunits that have regulatory (*pik3r1*/p85α, *pik3r1*/p55α, *pik3r1*/p50α, *pik3r2*/p85β and *pik3r3*/p55γ) and catalytic (*pik3ca*/p110α, *pik3cb*/p110β and *pik3cd*/p110 δ) functions. On the other hand, Class I_B_ consists of a single catalytic subunit—*pik3cg*/p110γ, and two regulatory subunits—*pik3r5/*p101 and—*pik3r6/*p84^13–16^. In response to specific stimuli, such as cytokines, these catalytic and regulatory subunits are activated and form heterodimers at the receptors through tyrosine-kinase-dependent mechanisms^16^. Association of catalytic subunits with the regulatory subunits is critical for the stabilization of the signaling complexes and for the recruitment of phospho-tyrosines on adaptor molecules.

Even though pioneering studies have established indispensable roles for specific catalytic subunits (p110δ, p110β and p110α) of Class I_A_ PI3K in immune cells^17–27^, functions of the regulatory subunits of Class I_A_ PI3K in the immune system, particularly in DCs, remain largely unknown. To date, there are only 3 studies reported on the involvement of the regulatory subunits in DCs. In the first two studies, through the use of germline p85α knockout (KO) mice, Koyasu and colleagues demonstrated the p85α subunit of Class I_A_ PI3K is essential for the suppression of IL12 secretion in DCs^28,29^. In the third study, Dalter et al. established that p85α subunit promotes pathogenic functions of DCs upon Experimental autoimmune encephalomyelitis (EAE) induction in mice^30^. While these studies elegantly demonstrated the importance of p85α subunit in DC activation, to date, functions of regulatory subunits of Class I_A_ PI3K in the development and differentiation of DCs remain completely unknown. More importantly, knowledge on the in-vivo dependence and roles of p85 subunits in the specification of cDC1, cDC2 and pDC lineages from hematopoietic stem cells (HSCs) remains elusive.

In an attempt to identify the previously unknown roles of p85 subunits in early DC development and in the differentiation of DC subsets, we deleted both p85α and β subunits of Class I_A_ PI3Ks in the hematopoietic system. Our studies, for the first time, demonstrate that a deficiency of p85 subunits causes an imbalance in the differentiation and maintenance of lymphoid-, myeloid- and plasmacytoid- DC subsets. Furthermore, our studies identify that p85α, but not p85β, subunit has an indispensable role in maintaining DC homeostasis of the primary and secondary lymphoid tissues.

## Results

### Loss of p85 results in increased numbers of DCs in the spleen

**Figure 1:**
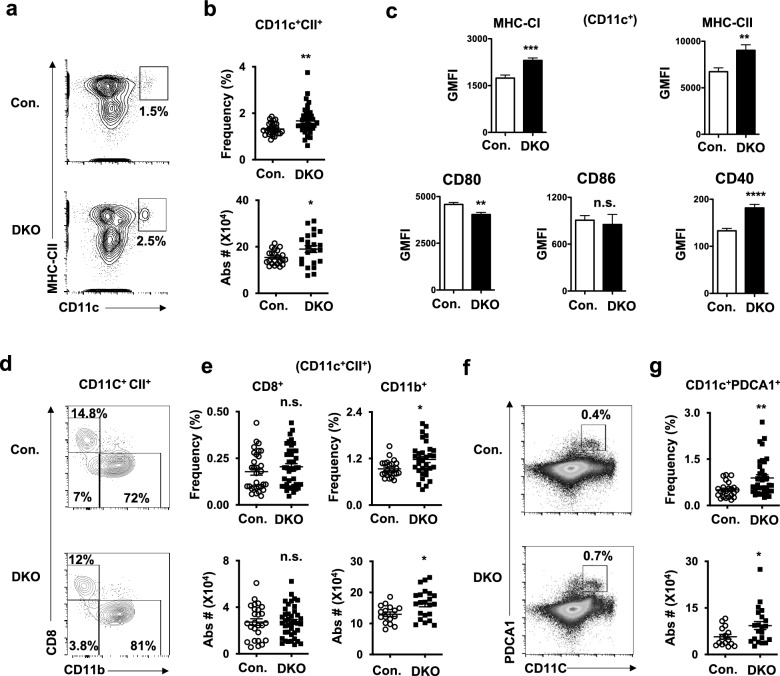
p85 deficiency leads to increased differentiation of DCs in the spleen. (**a**) FACS plots indicating frequencies of CD11c^+^CII^+^ DCs in the spleen of p85α and p85β (DKO) and control mice. Shown are the frequencies within the indicated gates. Data are representative of 5 independent experiments. (**b**) Frequencies (top) (n = 28–38) and absolute numbers (bottom) (n = 22) of CD11c^+^CII^+^ cells in the spleen of DKO and control mice. Indicated are the overall frequencies within spleen. (**c**) Surface expression levels of MHC-Class I, MHC-Class II, CD80, CD86 and CD40 in splenic CD11c^+^ cells of and control mice (n = 11). Shown are Geomean fluorescence Intensities (GMFI) of surface markers in CD11c^+^ gated cells. (**d**) FACS plots indicating frequencies of CD8^+^ (cDC1) and CD11b^+^ (cDC2) subsets within pre-gated CD11c^+^CII^+^ fraction in the spleen of p85α and p85β (DKO) and control mice. Shown are the frequencies within the indicated gates. Data are representative of 5 independent experiments. (**e**) Frequencies (top) (n = 31–40) and absolute numbers (bottom) (n = 16–40) of cDC1 (left) and cDC2 (right) subsets in the spleen of DKO and control mice. Indicated are the overall frequencies within spleen. (**f**) FACS plots indicating frequencies of CD11c^+^PDCA1^+^ (pDC) fraction in spleen of DKO and control mice. Data are representative of 5 independent experiments. Shown are the frequencies within the indicated gates. (**g**) Frequencies (top) (n = 23–36) and absolute numbers (bottom) (n = 15–21) of pDC subsets in the spleen of DKO and Control mice. Indicated are the overall frequencies within spleen. Data represent mean and s.e.m. Two-tailed student’s t tests were used to assess statistical significance (*P < 0.05, **P < 0.01, *** < 0.001, **** < 0.0001, ^n.s.^ *P* > 0.05).

Earlier studies established that p85α and p85β subunits compensate for the loss of each other in hematopoietic cells^31^. Thus, we decided to ablate both p85α and p85β subunits of PI3K to study their roles in DC differentiation. We crossed p85α^F/F^^32^ and p85β^−/−^^33^ mice to obtain p85α^F/F^p85β^−/−^ mice and these mice were crossed with Vav^cre/+^^34^ transgenic mice to generate p85α^F/F^p85β^−/−^Vav^cre/+^ mice (henceforth referred to as double knockout (DKO) mice), as expression of cre through Vav promoter results in faithful deletion of transgenes, exclusively, in hematopoietic organs and cells^35^. Our analysis of 4 weeks old DKO mice indicated normal frequencies of granulocytes (CD11b^+^Ly6G^+^), monocytes/macrophages (CD11b^+^Ly6G^−^), erythrocytes (Ter119^+^), megakaryocytes (CD41^+^) and B lymphocytes (CD19^+^) (Supplementary Fig. 1a–h). Next, we assessed the frequencies of bona-fide CD11c^+^CII^+^ DCs in the spleen and our data indicated an increased relative and absolute numbers of CD11c^+^CII^+^ total DCs in DKO mice (Fig. 1a,b).

To assess if Vav^cre^-mediated deletion of floxed alleles results in efficient recombination in all peripheral DC subsets, we generated and analyzed splenic DC subsets from Vav^Cre^Rosa-EGFP reporter mice. As shown in Supplementary Fig. 2a, > 97% of Total DCs (pool of cDC1 + cDC2 + pDC subsets), cDC1, cDC2 and pDCs exhibited expression of EGFP. These data unequivocally demonstrate that Vav^cre^-mediated recombination is very effective in all DC subsets. To further strengthen these findings and to directly prove that exon 7 of *Pi3kr1* (p85α) gene is efficiently deleted in DKO DCs, we sorted total DCs (CD11c^+^) from the bone marrow and spleen of DKO and Control mice and performed PCR studies, as described earlier^32^. Overall, our genomic PCR data (Supplementary Fig. 2b) and RT-PCR data (Supplementary Fig. 2c) indicated that floxed exon 7 of *Pi3kr1* gene is efficiently deleted in the DCs of DKO mice. Even though p85β is a (germline/conventional) total KO and the deletion of p85β is not dependent on Vav^cre^, we performed RT-PCR studies^31^ to detect deletion efficiency of *Pi3kr2* (p85β) gene in DKO DCs. Our data indicated that exon1 of *Pi3kr2* was completely deleted in DCs of both spleen and BM from DKO mice (Supplementary Fig. 2c). Taken together, these data demonstrate that both *Pi3kr1* and *Pi3kr2* genes are efficiently deleted in DKO DCs.

Immunophenotyping analysis indicated increased surface expression levels of MHC Class I, MHC Class II and CD40, decreased expression levels of CD80 and normal levels of CD86, in CD11c^+^ splenic DCs of DKO mice (Fig. 1c). Further fractionation of CD11c^+^CII^+^ DCs into CD8^+^CD11b^−^ cDC1and CD8^−^CD11b^+^ cDC2 subsets^4,6,7^, indicated normal frequencies and absolute numbers of cDC1, but augmented frequencies and absolute numbers of cDC2 in DKO spleen (Fig. 1d,e). Finally, enumeration of PDCA1^+^CD11c^+^ pDCs revealed an increase in both relative and absolute numbers of pDCs in the spleen of DKO mice (Fig. 1f,g). Taken together, these studies indicate that p85 mediated signals influence differentiation and/or maintenance of DC subsets in the spleen.

### Deficiency of p85 subunits reduces DC numbers in primary lymphoid organs

**Figure 2:**
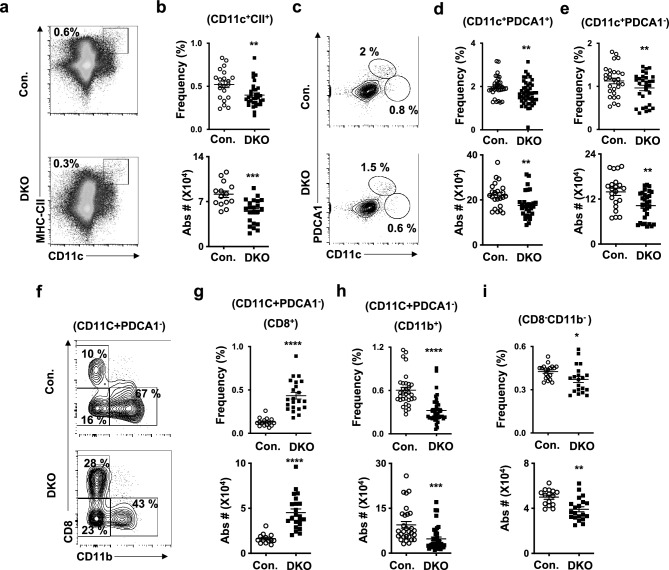
Loss of p85 causes reduced DC numbers in the BM. (**a**) FACS plots indicating frequencies of CD11c^+^CII^+^ DCs in the BM of DKO and control mice. Shown are the frequencies within the indicated gates. Data are representative of 5 independent experiments. (**b**) Frequencies (top) (n = 21–32) and absolute numbers (bottom) (n = 14–22) of CD11c^+^CII^+^ DCs in the BM of DKO and control mice. Indicated are the overall frequencies within BM. (**c**) FACS plots indicating frequencies of CD11c^+^PDCA1^+^ (pDC) and CD11c^+^PDCA1^−^ fractions in the BM of DKO and control mice. Shown are the frequencies within the indicated gates. Data are representative of 5 independent experiments. (**d**) Frequencies (top) (n = 34–47) and absolute numbers (bottom) (n = 23–32) of pDCs in BM of DKO and control mice. Indicated are the overall frequencies within BM. (**e**) Frequencies (top) (n = 25–30) and absolute numbers (bottom) (n = 21–30) of CD11c^+^PDCA1^−^ cells in the BM of DKO and control mice. Indicated are the overall frequencies within BM. (**f**) FACS plots indicating frequencies of CD8^+^, CD11b^+^ and CD8^−^CD11b^−^ subsets within CD11c^+^PDCA1^−^ fraction of BM of DKO and control mice. Shown are the frequencies within the indicated gates. Data are representative of 5 independent experiments. (**g**) Frequencies (top) (n = 15–22) and absolute numbers (bottom) (n = 13–22) of CD8^+^ cells within CD11c^+^PDCA1^−^ cells in the BM of DKO and control mice. Indicated are the overall frequencies within BM. (**h**) Frequencies (top) (n = 32–40) and absolute numbers (bottom) (n = 24–32) of CD11b^+^ cells within CD11c^+^PDCA1^−^ cells in the BM of DKO and control mice. Indicated are the overall frequencies within BM. (**i**) Frequencies (top) (n = 19–20) and absolute numbers (bottom) of CD8^−^CD11b^−^ (n = 15–20)cells within CD11c^+^PDCA1^−^ cells in BM of DKO and control mice. Indicated are the overall frequencies within BM. Data represent mean and s.e.m. Two-tailed student’s t tests were used to assess statistical significance (*P < 0.05, **P < 0.01, *** < 0.001, **** < 0.0001).

To investigate the DC compartments of the BM, we determined the frequencies of CD11c^+^CII^+^ cells. In contrast to the spleen, the frequencies and absolute numbers of CD11c^+^CII^+^ DCs are remarkably reduced in the BM of DKO mice (Fig. 2a,b). pDCs are continuously produced in the BM and represent the major DC fraction of the BM^36^ and our data indicated a reduction of both CD11c^+^ PDCA1^+^ pDCs and CD11c^+^ PDCA1^−^ cDCs in DKO BM (Fig. 2c–e). Discrimination of CD11c^+^PDCA1^−^ cDCs into cDC1 and cDC2 indicated an increase in frequencies and absolute numbers of CD8^+^ cDC1 (Fig. 2f,g) and decrease in frequencies and absolute numbers of CD11b^+^ cDC2 (Fig. 2f,h) subsets in the BM of DKO mice. Further analysis indicated reduced frequencies and absolute numbers of CD8^−^CD11b^−^ cells in the BM of DKO mice (Fig. 2f,i).

**Figure 3:**
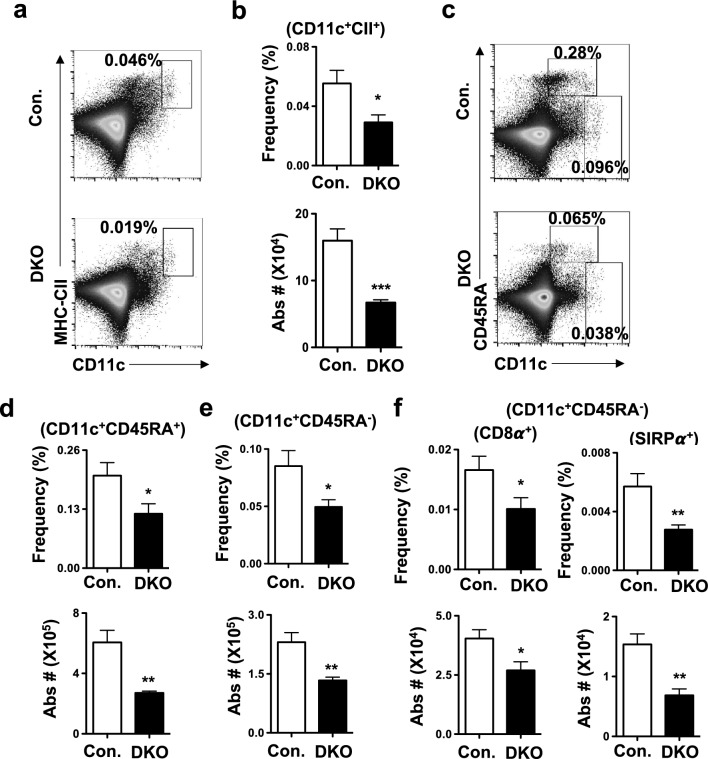
Deficiency of p85 subunits leads to reduced DC numbers in lymphoid organs. (**a**) FACS plots indicating frequencies of CD11c^+^CII^+^ DCs in the thymus of DKO and control mice. Data are representative of 5 independent experiments. Shown are the frequencies within the indicated gates. (**b**) Frequencies (top) and absolute numbers (bottom) of CD11c^+^CII^+^ DCs in the thymus of DKO and control mice (n = 8). Indicated are the overall frequencies within the organ. (**c**) FACS plots indicating frequencies of CD11c^+^CD45RA^+^ pDCs and CD11c^+^CD45RA^−^ cDCs in the thymus of DKO and control mice. Data are representative of 5 independent experiments. Shown are the frequencies within the indicated gates. (**d**) Frequencies (top) and absolute numbers (bottom) of CD11c^+^CD45RA^+^ pDCs in the thymus of DKO and control mice (n = 8). Indicated are the overall frequencies within the organ. (**e**) Frequencies (top) and absolute numbers (bottom) of CD11c^+^CD45RA^−^ pDCs in the thymus of DKO and control mice (n = 8). (**f**) Frequencies (top) and absolute numbers (bottom) of CD8α^+^ (cDC1) (left) and Sirpα^+^ (cDC2) (right) subsets within CD11c^+^CD45RA^−^ in the thymus of DKO and control mice (n = 8). Indicated are the overall frequencies within the organ. Data represent mean and s.e.m. Two-tailed student’s t tests were used to assess statistical significance (*P < 0.05, **P < 0.01, *** < 0.001, ^n.s.^ *P* > 0.05).

Next, we assessed the DC compartments in thymus of DKO mice. Our analysis indicated reduced frequencies and absolute numbers of overall CD11c^+^CII^+^ DCs in the thymus of DKO mice (Fig. 3a,b). Immunophenotyping studies on specific thymic DC subsets, following a previously established scheme^37^, revealed a reduction in both relative and absolute numbers of CD11c^+^CD45RA^+^ pDCs (Fig. 3c,d) and CD11c^+^CD45RA^−^ cDCs (Fig. 3c,e) in DKO mice. Further analysis of CD11c^+^CD45RA^−^ fraction indicated a reduction of CD8^+^ Sirpα^−^ cDC1 and CD8^−^ Sirpα^+^ cDC2 subsets in the thymus of DKO mice (Fig. 3f). Overall, these data indicate that p85 deficiency leads to an increase of cDC2 and pDCs in the spleen, but a global reduction of DCs in primary lymphoid organs.

### Lack of p85α subunit causes alterations in DC subsets

To identify specific functions of p85α vs. p85β subunit in DCs, we generated mice that are deficient for either p85α subunit (p85α ^F/F^Vav^cre^) or p85β subunit (p85β^−/−^). Analysis of spleen from 6 to 8 weeks old p85α ^F/F^Vav^cre^ mice indicated increased frequencies of cDCs (CD11c^+^CII^+^), cDC2 (CD11c^+^CII^+^CD11b^+^) subset and pDCs (CD11c^+^PDCA1^+^), and normal frequencies of cDC1 (CD11c^+^CII^+^ CD8^+^) subset (Supplementary Fig. 3a). On the other hand, analysis of spleen from 6 to 8 weeks old p85β^−/−^ mice revealed normal frequencies of cDCs, cDC1, cDC2 and pDCs (Supplementary Fig. 3b). Consistently, analysis of BM from 6 to 8 weeks old p85α ^F/F^Vav^cre^ mice indicated reduced frequencies of cDCs (CD11c^+^CII^+^ & CD11c^high^PDCA1^−^), pDCs (CD11c^+^PDCA1^+^), cDC2 (CD11c^+^PDCA1^−^CD11b^+^) subset, but increased frequencies of cDC1 (CD11c^+^PDCA1^−^CD8^+^) subset (Supplementary Fig. 3c). Finally, BM studies from 6 to 8 weeks old p85β^−/−^ mice revealed normal frequencies of cDCs, pDCs, cDC1 and cDC2 subsets (Supplementary Fig. 3d). Taken together, these data demonstrate that loss of p85α subunit alone recapitulates the phenotype of DKO mice and that p85β subunit has a dispensable role in DCs.

### Deletion of p85 subunits affects early stages of DC differentiation

**Figure 4:**
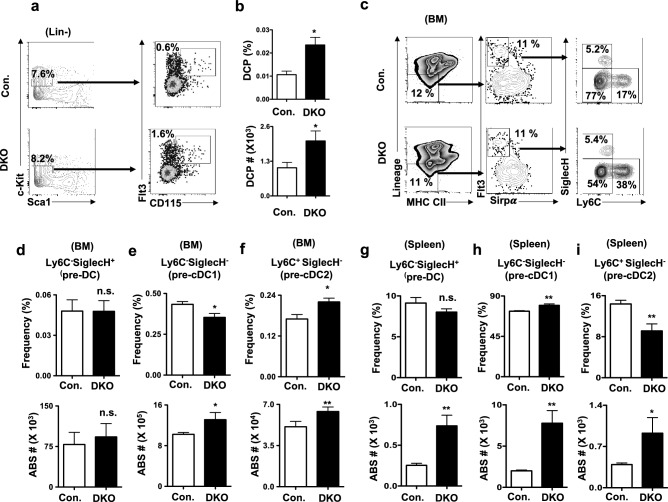
Deletion of p85 subunits affects early stages of DC differentiation. (**a**) FACS plots indicating frequencies of Lin^−^Sca1^−^c-Kit^int^Flt3^+^CD115^+^ CDPs in the BM of DKO and control mice. Cells were pre-gated on Lin^−^ cells of the BM. Shown are the frequencies within the indicated gates. Data are representative of 3 independent experiments. (**b**) Frequencies (top) and absolute numbers (bottom) of CDPs in BM of DKO and control mice (n = 11). Indicated are the overall frequencies within the organ. (**c**) FACS plots indicating frequencies of Ly6C^−^SiglecH^+^ (pre-DC), Ly6C^−^SiglecH^−^ (pre-cDC1) and Ly6C^+^SiglecH^−^ (pre-cDC2) subsets within Lin^−^CII^−^Flt3^+^Sirpα^−^ fraction in the BM of DKO and control mice. Shown are the frequencies within the indicated gates. Data are representative of 3 independent experiments. (**d**–**f**) Frequencies (top) and absolute numbers (bottom) of Ly6C^−^SiglecH^+^ (pre-DC) (**d**), Ly6C^−^SiglecH^−^ (pre-cDC1) (**e**) and Ly6C^+^SiglecH^−^ (pre-cDC2) (**f**) subsets within Lin^−^CII^−^Flt3^+^Sirpα^−^ fraction in the BM of DKO and control mice (n = 11). Indicated are the overall frequencies within the organ. (**g**–**i**) Frequencies (top) and absolute numbers (bottom) of Ly6C^−^SiglecH^+^ pre-DC (**g**), Ly6C^−^SiglecH^−^ pre-cDC1 (**h**) and Ly6C^+^SiglecH^−^ pre-cDC2 (**i**) subsets within Lin^−^CII^−^Flt3^+^Sirpα^+^ fraction in the spleen of DKO and control mice (n = 11). Indicated are the frequencies within the parent gates. Data represent mean and s.e.m. Two-tailed student’s t tests were used to assess statistical significance (*P < 0.05, **P < 0.01, ^n.s.^ *P* > 0.05).

Earlier studies established that common DC progenitors (CDPs) in the murine BM are predominantly restricted to the DC lineage and are precursors of cDC1, cDC2 and pDCs subsets. Moreover, DCs of majority of the non-lymphoid organs, such as lungs, liver, kidney skin and intestine, are believed to differentiate from CDPs^4,6,7^. To identify the functions of p85 in CDP generation and early stages of DC differentiation, we enumerated the frequencies of CDPs in the BM of DKO mice. CDPs within the mouse BM are identified as Lin^−^c-Kit^int^ Flt3^+^MCSFR^+^ cells^38^. Our analysis indicated that the frequencies and absolute numbers of CDPs were augmented in the BM DKO mice (Fig. 4a,b).

Next, we focused on the early developmental stages of DCs in the BM and spleen. Previous work proposed that CDPs differentiate into pre-DCs, pre-cDCs and committed precursors of cDC1 and cDC2 lineages^39^. According to this DC differentiation scheme^39^, late CDPs differentiate into Lin^−^CII^−^Sirpα^−^CD11c^+^Flt3^+^Siglec-H^+^Ly6C^−^ pre-DCs, that retain the potential to differentiate into cDC1, cDC2 and pDC lineages. These pre-DCs further differentiate into Lin^−^CII^−^Sirpα^−^CD11c^+^Flt3^+^Siglec-H^+^Ly6C^+^ pre-cDCs, that contain the potential to differentiate into cDC1 and cDC2, but lack pDC differentiation potential. In subsequent stages, pre-cDCs differentiate into either cDC1 lineage restricted Lin^−^CII^−^Sirpα^−^CD11c^+^Flt3^+^Siglec-H^−^Ly6C^−^ (pre-cDC1) or cDC2 lineage restricted Lin^−^CII^−^Sirpα^−^CD11c^+^Flt3^+^Siglec-H^−^Ly6C^+^ (pre-cDC2) subsets. Our analysis indicated normal frequencies of pre-DCs and pre-cDCs, decreased frequencies of pre-cDC1 and increased frequencies of pre-cDC2 subsets in the BM of DKO animals (Fig. 4c–f). On the other hand, analysis of spleen revealed normal frequencies of pre-DC (Fig. 4g), increased frequencies of pre-cDC1 (Fig. 4h) and decreased frequencies of pre-cDC2 (Fig. 4i) subsets in DKO mice. However, absolute numbers of pre-DC, pre-cDC1 and pre-cDC2 were higher in the spleen of DKO mice (Fig. 4g–i). In essence, these studies establish that p85 deficiency leads to abnormal DC developmental program in both BM and spleen.

### Absence of p85 subunits leads to augmented DC differentiation in-vitro

**Figure 5:**
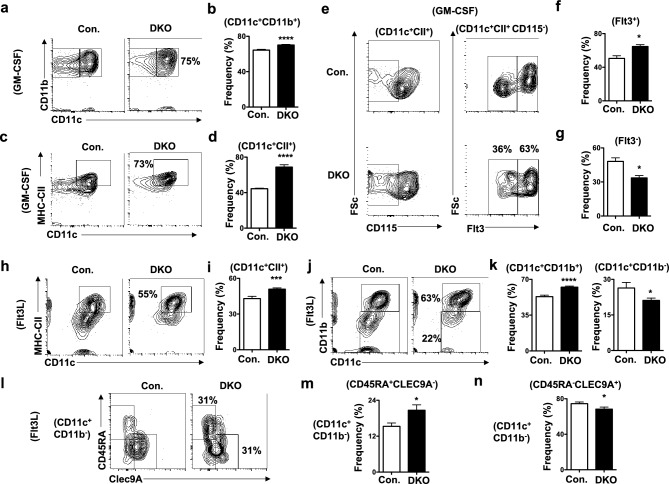
Deficiency of p85 subunits results in increased in-vitro DC differentiation. (**a**) FACS plots indicating frequencies of CD11b^+^CD11c^+^ cells from the BM of DKO and control mice cultured in-vitro in the presence of GM-CSF for 7 days. Shown are the frequencies within the indicated gates. (**b**) Frequencies of CD11b^+^CD11c^+^ cells from the BM of DKO and control mice cultured in-vitro in the presence of GM-CSF for 7 days (n = 3). Shown are the frequencies within the indicated gates. (**c**) FACS plots indicating frequencies of CD11c^+^CII^high^ cells from the BM of DKO and control mice cultured in-vitro in the presence of GM-CSF for 7 days. Shown are the frequencies within the indicated gates. (**d**) Frequencies of CD11c^+^CII^high^ cells from the BM of DKO and control mice cultured in-vitro in the presence of GM-CSF for 7 days (n = 3). Shown are the frequencies within the indicated gates. (**e**) FACS plots indicating frequencies of Flt3^+^ and Flt3^−^ cells from the BM of DKO and control mice cultured in-vitro in the presence of GM-CSF for 7 days (n = 3). Cells were pre-gated on CD11c^+^CII^high^ CD115^−^ cells. Shown are the frequencies within the indicated gates. (**f**) Frequencies of Flt3^+^ cells within CD11c^+^CII^high^ CD115^−^ fraction from the BM of DKO and control mice cultured in-vitro in the presence of GM-CSF for 7 days (n = 3). Shown are the frequencies within the indicated gates. (**g**) Frequencies of Flt3^−^ cells within CD11c^+^CII^high^ CD115^−^ fraction from the BM of DKO and control mice cultured in-vitro in the presence of GM-CSF for 7 days (n = 3). Shown are the frequencies within the indicated gates. (**h**) FACS plots indicating frequencies of CD11c^+^CII^+^ cells from the BM of DKO and control mice cultured in-vitro in the presence of Flt3L for 14 days. Shown are the frequencies within the indicated gates. (**i**) Frequencies of CD11c^+^CII^+^ cells from the BM of DKO and control mice cultured in-vitro in the presence of Flt3L for 14 days (n = 3). Shown are the frequencies within the indicated gates. (**j**) FACS plots indicating frequencies of CD11c^+^CD11b^+^ cells from the BM of DKO and control mice cultured in-vitro in the presence of Flt3L for 14 days. Shown are the frequencies within the indicated gates. (**k**) Frequencies of CD11c^+^CD11b^+^ (left) and CD11c^+^CD11b^−^ (right) cells from the BM of DKO and control mice cultured in-vitro in the presence of Flt3L for 14 days (n = 3). Shown are the frequencies within the indicated gates. (**l**) FACS plots indicating frequencies of CD45RA^+^Clec9A^−^ and CD45RA^−^Clec9A^+^ cells from the BM of DKO and control mice cultured in-vitro in the presence of Flt3L for 14 days. Cells were pre-gated on CD11c^+^CD11b^−^ cells. Shown are the frequencies within the indicated gates. (**m**) Frequencies of CD45RA^+^Clec9A^−^ cells within CD11c^+^CD11b^−^ fraction from the BM of DKO and control mice cultured in-vitro in the presence of Flt3L for 14 days (n = 3). Shown are the frequencies within the indicated gates. (**n**) Frequencies of CD45RA^−^Clec9A^+^ cells within CD11c^+^CD11b^−^ fraction from the BM of DKO and control mice cultured in-vitro in the presence of Flt3L for 14 days (n = 3). Shown are the frequencies within the indicated gates. Data represent mean and s.e.m. Two-tailed student’s t tests were used to assess statistical significance (*P < 0.05, **P < 0.01, *** < 0.001, **** < 0.0001, ^n.s.^ *P* > 0.05).

To test the differentiation capacities of p85 deficient BM into DCs, we performed in-vitro DC differentiation studies. First, total BM cells from DKO mice cultured in the presence of GM-CSF + IL4 for 7 days exhibited increased frequencies of CD11c^+^CD11b^+^ cells (Fig. 5a,b). Helft et al., documented that GM-CSF cultured BM cells give rise to both DCs and macrophages, and established a more refined immunophenotyping scheme to assess DC subsets generated under these conditions^40^. Following this scheme, we identified increased differentiation of DKO BM into CD11c^+^CII^high^ DCs (Fig. 5c,d). Further discrimination^40^ of CD11c^+^CII^high^ fraction of DKO group revealed an increase in frequencies of Flt3^+^CD115^−^ DCs (Fig. 5e,f) and a decrease in frequencies of Flt3^−^CD115^−^ cells (Fig. 5e,g). To assess the responses of GM-CSF cultured DCs to activation stimulus, we treated them with TNFα for 24 h. Analysis indicated normal surface levels of CD80 and CD40, but increased surface levels of CD86, in CD11c^+^CII^high^ DCs under unstimulated conditions (Supplementary Fig. 1i). However, surface expression levels of CD80, CD40 and CD86 were upregulated in DKO CD11c^+^CII^high^ DCs in response to TNFα (Supplementary Fig. 1j).

Next, we differentiated total BM cells in the presence of Flt3L for 14 days^41^. Consistent with the data of GM-CSF mediated DC differentiation, Flt3L cultured DKO BM resulted in increased frequencies of CD11c^+^CII^high^ DCs (Fig. 5h,i). Previous studies documented that BM cells cultured in the presence of Flt3L efficiently differentiate into DC subsets, that are considered as equivalents of cDC1, cDC2 and pDCs^41^. Following this immunophenotyping approach^41^, our analysis on Flt3L treated DKO BM cultures identified increased frequencies of CD11b^+^CD11c^+^ (cDC2 equivalent) DCs and reduced frequencies of CD11b^−^CD11c^+^ DC fraction (Fig. 5j,k). Further discrimination of CD11b^−^CD11c^+^ cells from DKO BM cultures identified an increased differentiation of CD45RA^+^Clec9A^−^ (pDC equivalent) (Fig. 5l,m) and a modestly decreased differentiation of CD45RA^−^Clec9A^+^ (cDC1 equivalent) (Fig. 5l,n) DC subsets.

Immunophenotyping analysis of DC associated surface markers revealed (Supplementary Fig. 1k); (1) an upregulation of MHC-CI in CD11b^+^, CD45RA^+^ and Clec9A^+^ DC subsets; (2) an upregulation of MHC-CII in CD11b^+^ and Clec9A^+^, and normal levels in CD45RA^+^, DC subsets; (3) an upregulation of CD80 in CD11b^+^, but normal levels in CD45RA^+^ and Clec9A^+^, DC subsets; (4) a down regulation of CD86 in CD11b^+^, an upregulation of CD86 in CD45RA^+^, and normal levels of CD86 in Clec9A^+^, DC subsets; and (5) an upregulation of CD40 in CD11b^+^, CD45RA^+^ and Clec9A^+^ DC subsets. Together, these in-vitro DC differentiation studies suggest that p85 deficiency causes augmented differentiation of myeloid DCs in the presence of DC promoting cytokines.

### p85 deficiency affects DC differentiation through cell-intrinsic pathways

**Figure 6:**
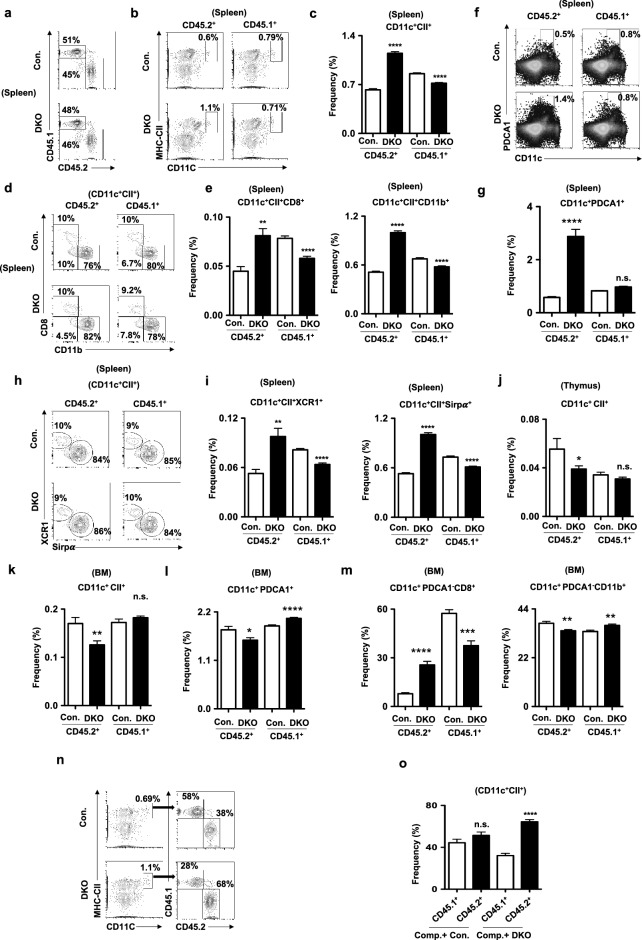
DC phenotype of p85 deficient mice is caused by cell intrinsic mechanisms. (**a**) FACS plots indicating frequencies of either control or DKO (CD45.2^+^) and wildtype competitor (CD45.1^+^**)** derived hematopoiesis in the spleen of lethally irradiated wildtype congenic (CD45.1^+^**)** recipients after 8 weeks of mixed BMT. Shown are the frequencies within the indicated gates. (**b**) FACS plots indicating frequencies of either control or DKO (CD45.2^+^) and wildtype competitor (CD45.1^+^**)** derived DCs (CD11c^+^CII^+^) in the spleen of BMT recipients. Shown are the frequencies within the indicated gates. (**c**) Frequencies of either control or DKO (CD45.2^+^) and wildtype competitor (CD45.1^+^**)** derived DCs in the spleen of mixed BMT recipients (n = 9–15) after 8 weeks. Indicated are the overall frequencies within the organ. (**d**) FACS plots indicating frequencies of either control or DKO (CD45.2^+^) and wildtype competitor (CD45.1^+^**)** derived cDC1 (CD8^+^CD11c^+^CII^+^) and cDC2 (CD11b^+^CD11c^+^CII^+^) subsets in the spleen of BMT recipients after 8 weeks. Shown are the frequencies within the indicated gates. (**e**) Frequencies of either control or DKO (CD45.2^+^) and wildtype competitor (CD45.1^+^**)** derived cDC1 (CD8^+^CD11c^+^CII^+^) (left) and cDC2 (CD11b^+^CD11c^+^CII^+^) (right) subsets in the spleen of BMT recipients (n = 9–15) after 8 weeks. Indicated are the overall frequencies within the organ. (**f**) FACS plots indicating frequencies of either control or DKO (CD45.2^+^) and wildtype competitor (CD45.1^+^**)** derived pDCs (CD11c^+^PDCA1^+^) in the spleen of BMT recipients after 8 weeks. Shown are the frequencies within the indicated gates. (**g**) Frequencies of either control or DKO (CD45.2^+^) and wildtype competitor (CD45.1^+^**)** derived pDCs (CD11c^+^PDCA1^+^) in the spleen of BMT recipients (n = 9–15) after 8 weeks. Indicated are the overall frequencies within the organ. (**h**) FACS plots indicating frequencies of either control or DKO (CD45.2^+^) and wildtype competitor (CD45.1^+^**)** derived cDC1 (XCR1^+^Sirpα^−^ CD11c^+^CII^+^) and cDC2 (XCR1^−^Sirpα^+^CD11c^+^CII^+^)subsets in the spleen of BMT recipients after 8 weeks. Shown are the frequencies within the indicated gates. (**i**) Frequencies of either control or DKO (CD45.2^+^) and wildtype competitor (CD45.1^+^**)** derived cDC1 (XCR1^+^Sirpα^−^ CD11c^+^CII^+^) (left) and cDC2 (XCR1^−^Sirpα^+^CD11c^+^CII^+^) (right) subsets in the spleen of BMT recipients (n = 9–15) after 8 weeks. Indicated are the overall frequencies within the organ. (**j**) Frequencies of either control or DKO (CD45.2^+^) and wildtype competitor (CD45.1^+^**)** derived DCs (CD11c^+^CII^+^) in the thymus of BMT recipients (n = 9–15) after 8 weeks. Indicated are the overall frequencies within the organ. (**k**) Frequencies of either control or DKO (CD45.2^+^) and wildtype competitor (CD45.1^+^**)** derived DCs (CD11c^+^CII^+^) in the BM of BMT recipients (n = 9–15) after 8 weeks. Indicated are the overall frequencies within the organ. (**l**) Frequencies of either control or DKO (CD45.2^+^) and wildtype competitor (CD45.1^+^**)** derived pDCs (CD11c^+^PDCA1^+^) in the BM of BMT recipients (n = 9–15) after 8 weeks. Indicated are the overall frequencies within the organ. (**m**) Frequencies of either control or DKO (CD45.2^+^) and wildtype competitor (CD45.1^+^**)** derived cDC1 (CD11c^+^PDCA1^−^CD8^+^CD11b^−^) (left) and cDC2 (CD11c^+^PDCA1^−^CD8^−^CD11b^+^) (right) subsets in the BM of BMT recipients (n = 9–15) after 8 weeks. Indicated are the overall frequencies within the organ. (**n**) FACS plots indicating frequencies of total CD11c^+^CII^+^ cDCs (left) and frequencies of wildtype competitor (CD45.1^+^**)** derived and either control or DKO (CD45.2^+^) derived cells within cDCs of the spleen from same animal, respectively, after 8 weeks. (**o**) Frequencies of wildtype competitor (CD45.1^+^**)** derived and either control or DKO (CD45.2^+^) derived CD11c^+^CII^+^ cDCs of the spleen from same animal, respectively, after 8 weeks (n = 9–15). Data represent mean and s.e.m. Two-tailed student’s t tests were used to assess statistical significance (*P < 0.05, **P < 0.01, *** < 0.001, *** < 0.001, ^n.s.^ *P* > 0.05).

To investigate if the DC phenotype of p85 mutant mice is cell intrinsic, we performed mixed BMT assays^42^. Total BM cells from either control or DKO mice (CD45.2^+^) were mixed with total BM cells of wildtype congenic mice (CD45.1^+^), at a ratio of 1:1, and injected into lethally irradiated congenic (CD45.1^+^) recipients. Analysis of spleen (Fig. 6a) after 8 weeks of transplantation revealed; (1) an increase of DKO DCs (CD45.2^+^CD11c^+^CII^+^), when compared to both control DCs (CD45.2^+^CD11c^+^CII^+^) and wildtype competitor DCs (CD45.1^+^CD11c^+^CII^+^) (Fig. 6b,c); (2) increased frequencies of both CD8^+^ and CD11b^+^ DC subsets within DKO DCs (CD45.2^+^CD11c^+^CII^+^) (Fig. 6d,e); (3) increased frequencies of DKO pDCs (CD45.2^+^CD11c^+^PDCA1^+^) (Fig. 6f,g); and (4) increased frequencies of both XCR1^+^ and Sirpα^+^ DC subsets within DKO DCs (CD45.2^+^CD11c^+^CII^+^) (Fig. 6h,i). Consistent with the phenotype of DKO mice, mixed BM transplantation studies revealed reduced frequencies of DKO DCs (CD45.2^+^CD11c^+^CII^+^) in the thymus (Fig. 6j) and BM (Fig. 6k). Even though the frequencies of either control DCs (CD45.2^+^CD11c^+^CII^+^) or wildtype competitor DCs (CD45.1^+^CD11c^+^CII^+^) were normal within the same niche (Fig. 6j,k). Further analysis of BM indicated reduced frequencies of DKO pDCs (CD45.2^+^PDCA1^+^CD11c^+^) (Fig. 6l) and DKO cDC2 (CD45.2^+^PDCA1^−^CD11c^+^CD11b^+^) subset (Fig. 6m), whereas increased frequencies of DKO cDC1 (CD45.2^+^PDCA1^−^CD11c^+^CD8^+^) subset (Fig. 6m). To further strengthen these findings, we gated total CD11c^+^CII^+^ cDCs of the spleen from recipients and determined the DC differentiation capacities of wildtype competitor vs. donor derived progenitors under competitive settings within the same niche. Data indicated that the frequencies of cDCs derived from competitor and control donor were comparable (Fig. 6n,o). However, the frequencies of cDCs derived from DKO donor were increased, when compared with the same of the competitor (Fig. 6n,o). Overall, our mixed BM chimera studies established that the DC phenotype of DKO mice is caused by cell intrinsic defects in the absence of p85.

### Lack of p85 leads to augmented DC differentiation and defective signaling in DCs

**Figure 7:**
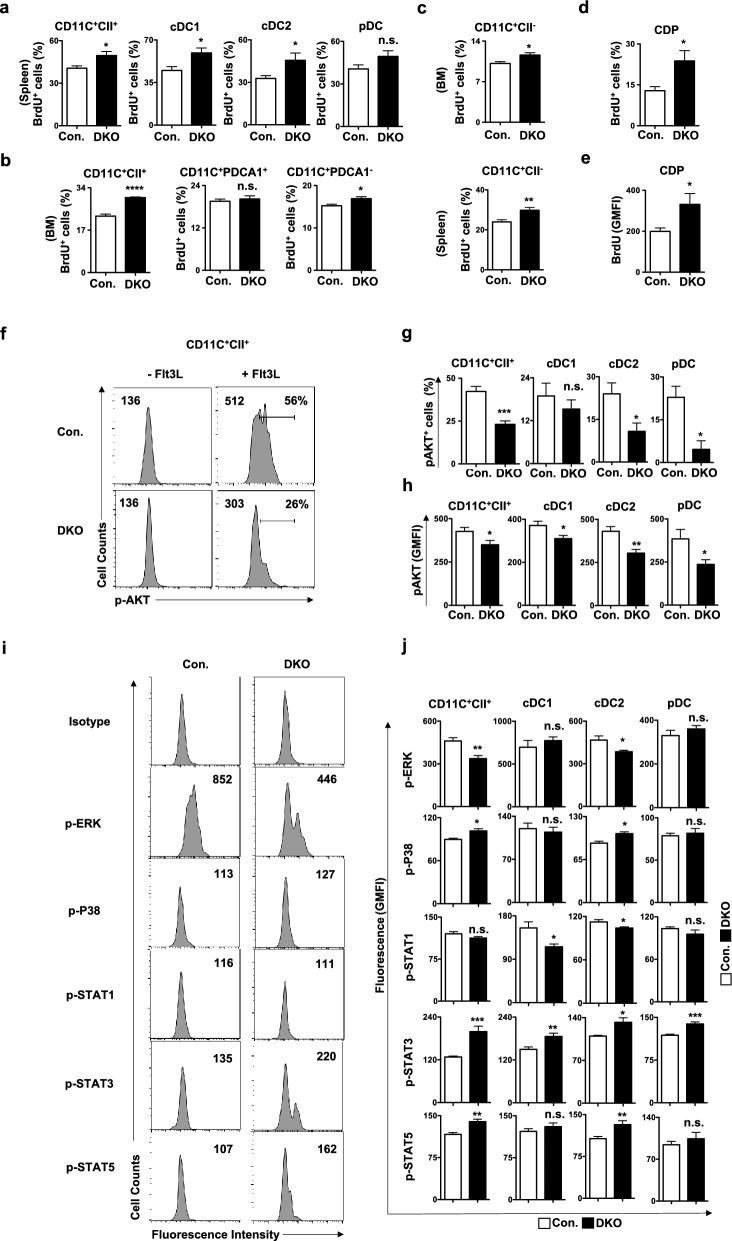
Lack of p85 subunits causes augmented proliferation and altered signaling in DCs. (**a**) Frequencies of BrdU^+^ cells within pre-gated CD11c^+^CII^+^, cDC1, cDC2 and pDC subsets of spleen from DKO and control mice (n = 8). Indicated are the frequencies within the parent gate. (**b**) Frequencies of BrdU^+^ cells within pre-gated CD11c^+^CII^+^, CD11c^+^PDCA1^−^ and CD11c^+^PDCA1^+^ subsets of BM from DKO and control mice (n = 7–8). Indicated are the frequencies within the parent gate. (**c**) Frequencies of BrdU^+^ cells within pre-gated CD11c^+^CII^−^ cells of BM (top) and spleen (bottom) from DKO and control mice ((n = 7–8). Indicated are the frequencies within the parent gate. (**d**) Frequencies of BrdU^+^ cells within pre-gated CDPs of BM from DKO and control mice (n = 7–8). (**e**) GMFI of BrdU within pre-gated CDPs of BM from DKO and control mice (n = 8). Indicated are the frequencies within the parent gate. (**f**) Histograms indicating levels of phospho-AKT (Thr308) in CD11c^+^CII^+^ DCs of control and DKO spleen after stimulation with Flt3L. Cells stimulated in the absence of Flt3L served as negative controls. Shown are the total GMFI (left) and frequencies (within the parent gate) of cells under the gate (right). (**g**) Frequencies of phospho-AKT (Thr308)^high^ cells within pre-gated CD11c^+^CII^+^, cDC1, cDC2 and pDC subsets of spleen from DKO and control mice (n = 8). Indicated are the frequencies within the parent gate. (**h**) GMFI of phospho-AKT (Thr308) within pre-gated CD11c^+^CII^+^, cDC1, cDC2 and pDC subsets of spleen from DKO and control mice (n = 8). (**i**) Histograms indicating levels of phospho-proteins in CD11c^+^CII^+^ DCs of control and DKO spleen after stimulation with Flt3L. Cells treated with isotype controls served as negative controls. Shown are the total GMFI (right). (**j**) GMFI of phospho-ERK, phospho-p38, phospho-STAT1, phospho-STAT3 and phospho-STAT5 within pre-gated CD11c^+^CII^+^, cDC1, cDC2 and pDC subsets of spleen from DKO and control mice (n = 8). Data represent mean and s.e.m. Two-tailed student’s t tests were used to assess statistical significance (*P < 0.05, **P < 0.01, *** < 0.001, *** < 0.001, ^n.s.^ *P* > 0.05).

To investigate mechanisms leading to abnormal DC differentiation and maintenance, we quantified proliferation of DC subsets and their precursors through BrdU incorporation assays^43^ and signal transduction pathways through phosflow technique^44^. Our BrdU assays indicated increased proliferation of CD11c^+^CII^+^ DCs in DKO spleen (Fig. 7a). DC subset analysis indicated increased proliferation of cDC1 and cDC2 subsets, but normal proliferation of pDCs, in the DKO spleen (Fig. 7a). Consistently, BM DC subsets indicated increased proliferation of CD11c^+^CII^+^ total DCs and CD11c^+^PDCA1^−^ cDCs, whereas normal proliferation rates of CD11c^+^PDCA1^+^ pDCs, in DKO BM (Fig. 7b). Proliferation studies on precursors revealed augmented proliferation rates of CD11c^+^CII^−^ DC precursors in both BM and spleen of DKO mice (Fig. 7c). BrdU quantification in Lin^−^c-Kit^int^Flt3^+^CD115^+^ CDPs documented enhanced proliferation rates of CDPs in the DKO BM (Fig. 7d,e). These data suggest that a deficiency of p85 subunits leads to increased proliferation of cDCs and augmented DC differentiation program due to hyperproliferation of CDPs and precursor DCs.

To understand the physiological changes caused by p85 deficiency in DCs, we quantified phosphorylation of proteins, involved in major signal transduction pathways, in DC subsets through phosflow analysis^44^. PI3Ks generate lipid-based second messengers that participate in a wide range of signaling pathways in response to stimulation by growth factors and cytokines^16^. Within the DC lineage, PI3Ks has been shown to be a major transducer of the signaling cascade initiated by Flt3L^45^. As expected, our phosflow analysis indicated reduced phosphorylation of AKT in total CD11c^+^CII^+^ DCs, as well as in cDC1, cDC2, pDC subsets, of DKO spleen in response to Flt3L (Fig. 7f–h). Quantification of Flt3L mediated phosphorylation levels of Erk, p38, STAT1, STAT3 and STAT5 signaling proteins in splenic DKO DC subsets revealed (Fig. 7i,j); (1) Erk phosphorylation was reduced, whereas p38 phosphorylation was increased, in total CD11c^+^CII^+^ DCs and cDC2 subset; (2) Phosphorylation of STAT1 was reduced in cDC1 and cDC2 subsets; (3) STAT3 phosphorylation was augmented in in total CD11c^+^CII^+^ DCs, as well as in cDC1, cDC2, pDC subsets; and (4) Phosphorylation levels of STAT5 were increased in total CD11c^+^CII^+^ DCs and cDC2 subset. Taken together, our phosflow studies reveal that loss of p85 leads to alterations in major signal transduction pathways in either total DCs or selective DC subsets.

## Discussion

In this study, we demonstrated that absence of both p85α and p85β subunits of PI3K causes deregulated differentiation of DCs. Our data show that loss of p85 subunits causes increased numbers of DCs in the spleen, decreased DC numbers in the BM, thymus and peripheral lymph nodes and mesenteric lymph nodes. In-vitro studies indicated that p85 deficiency causes increased differentiation of BM cells into DCs, both in the presence of GM-CSF and Flt3L. These observations are further corroborated by the fact that the numbers and proliferative properties of CDPs in the BM and precursors DCs in the spleen are increased in absence of p85α and β. These findings unequivocally demonstrate that p85 acts as a negative regulator of DC differentiation and proliferation. Interestingly, within the cDC compartment, p85 deficiency appears to differentially affect cDC1 and cDC2 development in lymphoid tissues. Of note, cDC1 numbers were normal in the spleen but increased in the BM, whereas cDC2 numbers were increased in the spleen and decreased in the BM. These differences in p85 mutant cDC1 and cDC2 subsets may be caused by the changes in the phosphorylation levels of signal transducers, including AKT, p38, ERK and STAT proteins, and/or functions of p85 subunits in cDC1 and cDC2 subsets in response to cytokines and growth factors. Consistent with this notion, deficiency of PI3Kγ causes severe loss of CD103^+^DCs in the lungs, but not in liver, small intestine, skin and kidneys^21^.

The numbers of cDC2 subset and pDCs are increased in the spleen, even though their numbers in other lymphoid organs are reduced. These findings present an interesting discrepancy and highlight a key role for p85 in the maintenance of DC pool in different tissues. According to the current understanding of DC development, precursor- cDCs and pDCs are generated from CDPs in the BM and enter the blood stream to seed several lymphoid and non-lymphoid tissues^46^. After reaching these tissues, pre-cDCs locally differentiate into mature cDC1 and cDC2. Based on this model, it can be speculated that p85 deficient pre-cDCs and pDCs preferentially migrate to the spleen and exhibit reduced migration to thymus or pLN. In support of this notion, earlier report established that a loss of PI3Kγ causes defective DC migration to skin and lymph nodes^18^. Our data indicated largely normal DC numbers in non-lymphoid organs, therefore suggesting that migration of pre-DCs to non-lymphoid organs seems to be intact in the absence of p85. However, it is unclear from the current studies if p85 deficiency causes any migration defects in DCs and this remains to be investigated.

Importance of PI3K signaling in the immune cells has been well recognized in T cell and B cell development. p85α, p85β, p110α and p110β isoforms are ubiquitously expressed, whereas p110δ and p110γ isoforms are preferentially expressed at higher levels in hematopoietic cells^16^. Loss of p110α leads to complete embryonic lethality and inactivation of p110β results in partial embryonic death^47,48^. Genetic studies conducted through either deletion of p110δ or transgenic expression of catalytically inactive form of p110δ (p110δ^D910A^) identified a crucial for p110δ subunit in B cell -development and -activation, B and T cell antigen receptor signaling and the restriction of autoimmune diseases^17,20,22^. In contrast, genetic deletion of p110γ resulted in defective thymocyte development, T cell activation, neutrophil/macrophage migration and functions, and increased systemic inflammation^19,23^.

Studies focused on the functions of class I_B_ PI3Ks in DCs concluded that p110γ deficiency causes; (1) reduced numbers of CD8α^+^ DCs and CD4^+^ DCs, and normal numbers of pDCs in the spleen; (2) reduced numbers of CD103^+^ DCs and CD11b^+^ DCs in the thymus; (3) reduced numbers of CD103^+^ DCs and CD11b^+^ DCs, and normal numbers of pDCs in the lungs; (4) reduced frequencies of CD103^+^ DCs and normal frequencies of CD11b^+^ DCs in lung draining lymph node; and 5 reduced frequencies of CD8α^−^DCs and normal frequencies of CD8α^+^ and pDCs in the pLNs^18,21^. Overall, these studies established an unequivocal role for the class I_B_ PI3Ks in the development of DCs. On the other hand, importance of class I_A_ PI3Ks in DC development is unclear.

Earlier studies using mice deficient for the catalytic subunit of class I_A_ PI3Ks concluded that P110δ deficiency does not affect DC differentiation and the loss of either p110α and p110β does not affect functions of DCs^24^. Consistently, Guiducci et al., reported that inhibition of P110δ does not affect human pDC differentiation^49^. In the present study, we ablated two key regulatory subunits—p85α and p85β of class I_A_ PI3Ks and studied their impact on DC differentiation. Data presented here, for the first time, unequivocally establish that the regulatory subunits of class I_A_ PI3Ks play pivotal roles in the development and maintenance of DCs. Even though our mechanistic studies provide novel insights on signal transduction pathways that might be responsible for the DC phenotype of p85 deficient mice, we believe that additional mechanisms might be involved in the regulation of DC subsets. Nevertheless, our studies highlight and establish a previously unknown function for class I_A_ PI3Ks in DC biology.

## Materials and methods

### Ethics statement

This study was carried out in accordance with the “*Guide for the Care and Use of Laboratory Animals*” as promulgated by the National Institute of Health and the protocols were approved by the Institutional Animal Care and Use Committees (IACUC) of Columbia University Medical Center and University of Maryland School of Medicine. All studies involving animals are reported in accordance with the *ARRIVE guidelines* for reporting experiments involving animals^50^.

### Mice

p85α^loxP^ (*Pik3r1*^*tm1Lca*^/J), p85β^−/−^ (*Pik3r2*^*tm1Lca*^/J), Vav-iCre (B6.Cg-Commd10^Tg(Vav1-icre)A2Kio^/J) Rosa^EGFP^ mice were purchased from the Jackson Laboratory. p85α^loxP^ mice^32^ harbor *loxP* sites flanking exon 7 (the first common exon for the three encoded isoforms (p85α, p55α, and p50α)) of the phosphatidylinositol 3-kinase, regulatory subunit, polypeptide 1 (p85 alpha) locus. Therefore, cre mediated deletion of p85 results in loss of all there isoforms in these mice. On the other hand, p85β mutant mice^33^ harbor a "knockout" allele of the phosphatidylinositol 3-kinase, regulatory subunit, polypeptide 2 (p85 beta) locus. Our immunophenotyping analysis indicated no difference among mice with Pi3kr1^F/F^, Pi3kr1^F/+^, and Pi3kr1^+/+^Vav ^Cre/+^ genotypes. Therefore, all these genotypes were used, whenever available, and referred to as “controls” in our DC studies. Of note, no differences in the DC subsets were observed from the BM (Supplementary Fig. 4) and spleen (Supplementary Fig. 5) of Pi3kr1^F/+^Vav ^Cre/+^ (Het) and control mice. Mice were maintained under specific pathogen–free conditions.

### Cell preparation

Mice were analyzed between 4 and 12 weeks after birth, unless otherwise specified.

Spleen and thymus were digested with Collagenase-D (0.7 mg/mL) and DNase I (100U/ mL) in RPMI for 45 min at 37° C. BM cells were isolated from the tibias and femurs by inserting a 23-gauge needle/1 mL syringe to the bone cavities and flushed with PBS 2%FCS until the bones become pale. Single cell suspensions were made through rigorous pipetting.

Red blood cells were lysed with Ammonium chloride (Stem Cell Technology) and subsequently filtered using a 70 nM nylon mesh. Bone marrow cells were then counted with a hemacytometer and trypan blue (Amresco) negative cells were counted as live cells.

### Flow cytometry

Cells were analyzed by flow cytometry with Attune Nxt (Thermofisher) and FlowJo software (Tree Star). The following monoclonal antibodies were used: anti- CD34 (RAM34), anti-CD48 (HM48-1), anti-CD117 (2B8), anti-Flt3 (A2F10.1), anti-Sca-1 (D7), anti-B220 (RA3-6B2), anti- CD19 (1D3), anti-CD3 (145-2C11), anti-CD4 (GK1.5), anti-CD8 (53–6.7), anti-CD11b (M1/70), anti– Gr-1 (RB6-8C5), anti-Ter119 (TER119), anti-CD11c (N418), anti-CD8(53–6.7), anti-PDCA1 (129C1), anti-CD24 (M1/69), anti-SIRPa (P84), anti-CD103 (2E7), anti-CD45 (S18009F), anti-CD207 (4C7), anti-CD115 (AFS98), anti-LY6C (HK1.4), anti-SiglecH (551), anti-CLEC9A (7H11), anti-CD80 (16-10A1), anti-CD86 (GL-1), anti-CD40(3/23), anti-H2K^b^ (AF6-88.5), anti-IA/IE (M5/114.15.2), anti-CD26 (H194-112), anti-XCR1 (Zet) and anti-BrdU (3D4) from Biolegend. Anti-pAKT (M89-61), anti-pERK (20A), anti-pP38 (36/p38), anti-pSTAT1 (4a), anti-pSTAT3(4/P-STAT3), anti-pSTAT5 (47/STAT5(pY694)) and anti-CD45RA (14.8) were purchased from BD Biosciences. Cells incubated with biotinylated monoclonal antibodies were incubated with fluorochrome-conjugated streptavidin–peridinin chlorophyll protein–cyanine 5.5 (551419; BD), streptavidin-allophycocyanin-Cy7 (554063; BD), streptavidin-super bright 650 (Biolegend). In all the FACS plots, indicated are the percentages (%) of the gated fraction. All antibodies were used at a concentration of 0.2 µg per 10^6^ cells in 100 µl staining volume. In all FACS plots, dead cells and debris were excluded based on Forward Scatter (FSc) and Side Scatter (SSc) properties, doublets were excluded based on FSc-H vs. FSc-A and SSc-H vs. SSc-A and fluorescence properties of pre-gated singlets were determined (Supplementary Fig. 6).

### In-vitro DC differentiation assays

4 × 10^6^ BM cells per well were cultured in 6 well pates in 4 ml of complete medium (DMEM supplemented with glutamine, penicillin, streptomycin, 2-mercaptoethanol [all from Invitrogen]), 10% heat-inactivated fetal calf serum and GM-CSF + IL4 (20 ng/ml each, Peprotech) at 37 °C with 5% CO_2_. Once in every 3 days, half of the medium was removed and replaced with fresh medium supplemented with GM-CSF + IL4. On day 7, cells were harvested, washed with ice cold PBS and analyzed by flow cytometry. For activation studies, 7-day cultured cells were further treated for 24 h in the presence of TNFα (100 ng/ml, Peprotech).

For Flt3L mediated DC differentiation studies, 4 × 10^6^ BM cells per well were cultured in 6 well pates in 4 ml of complete medium (DMEM supplemented with glutamine, penicillin, streptomycin, 2-mercaptoethanol [all from Invitrogen]), 10% heat-inactivated fetal calf serum and Flt3L (100 ng/ml, Peprotech) at 37 °C with 5% C0_2_. Once in every 3 days, half of the medium was removed and replaced with fresh medium supplemented with Flt3L. On day 14, cells were harvested, washed with ice cold PBS, surface stained and analyzed by flow cytometry.

### Cell proliferation assay

For in-vivo bromodeoxyuridine (BrdU) assays, 1 mg BrdU (BD) was injected intraperitoneally. After 24 h of injection, mice were sacrificed and bone marrow cells and splenocytes were surface stained with antibodies. Cells were fixed and permeabilized using BrdU Flow Kit manufacturer's instructions (BD Pharmingen) and stained with anti-BrdU for 1 h and analyzed by flow cytometry.

### Bone marrow transplantation (BMT) experiments

For mixed BM chimera studies, 5 × 10^5^ BM cells from either control or DKO mice were mixed with 5 × 10^5^ of WT (CD45.1^+^) BM cells (to obtain a ratio of 1:1) and were injected into lethally irradiated (10 Gy) congenic WT (CD45.1^+^) recipient mice. Mice were analyzed 8 weeks after transplantation.

### Phosflow studies

To detect phosphorylation of proteins by flow cytometry, cells were either stimulated with Flt3L or unstimulated for 30 min. Cells were surface stained, fixed and permeabilized using a commercially available phosflow kit (BD Pharmigen) according to the manufacturer’s instructions. Intra-cellular phospho- proteins were stained with PE-conjugated antibodies (BD biosciences) for 1 h and analysed by flow cytometry.

### RNA extraction, PCR, and real-time PCR

Total CD11c^+^ DCs of BM and spleen were sorted using Magnetic associated cell sorting (MACS) (Miltenyi Biotec). Genomic DNA and Total RNA was isolated with QIAamp DNA mini kit and RNeasy Mini kit (QIAGEN), respectively. cDNA was synthesized with Oligo(dT) primer and Superscript IV Reverse Transcriptase (Thermo Fisher Scientific). PCR was performed with T100 thermal cycler (Bio-Rad Laboratories) and TSG Taq (Lamda Biotech), using *Pik3r1* or *Pik3r2* deletion specific primers, as described earlier^31,32^. Relative expression of p85 cDNA was normalized to the expression levels of the internal control (housekeeping gene) HPRT.

### Statistics

Data represent mean and s.e.m. Two-tailed student’s t tests were used to assess statistical significance (*P < 0.05, **P < 0.01, *** < 0.001, **** < 0.0001).

## Supplementary Information

Supplementary Information 1.

Supplementary Information 2.

Supplementary Information 3.

## Data Availability

The datasets used and/or analyzed during the current study available from the corresponding author on reasonable request.
