# Correction: Class I PI3K regulatory subunits control differentiation of dendritic cell subsets and regulate Flt3L mediated signal transduction

**DOI:** 10.1038/s41598-025-03629-w

**Published:** 2025-06-25

**Authors:** Keyur Thummar, Chozha Vendan Rathinam

**Affiliations:** 1 Department of Genetics and Development, Columbia University Medical Center, New York, NY 10032 USA; 2 Institute of Human Virology, University of Maryland School of Medicine, 725 West Lombard Street, Baltimore, MD 21201 USA; 3 Center for Stem Cell and Regenerative Medicine, University of Maryland School of Medicine, 725 West Lombard Street, Baltimore, MD 21201 USA

Correction to: *Scientific Reports* 10.1038/s41598-022-16548-x, published online 19 July 2022

This Article contains an error in Fig. 6(f). Due to the error during the figure assembly, the image for the representative FACS plot shown in Fig. 6(f) was duplicated. The FACS plot that corresponds to control sample (indicated by yellow arrow) on both control and DKO panels was pasted by mistake. For the DKO group, we planned to paste the FACS plot that is indicated by blue arrow, however, erroneously pasted the control plot (yellow arrow). This change does not affect the conclusions of the Article.

The original Fig. 6 and accompanying legend appear below.

**Fig. 6:**
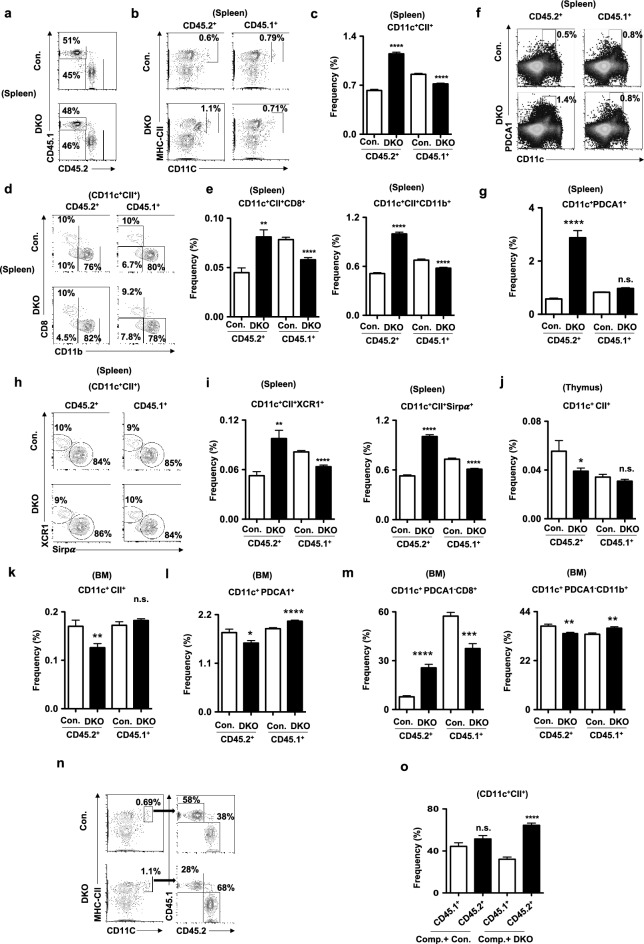
DC phenotype of p85 deficient mice is caused by cell intrinsic mechanisms. (**a**) FACS plots indicating frequencies of either control or DKO (CD45.2^+^) and wildtype competitor (CD45.1^+^) derived hematopoiesis in the spleen of lethally irradiated wildtype congenic (CD45.1^+^) recipients after 8 weeks of mixed BMT. Shown are the frequencies within the indicated gates. (**b**) FACS plots indicating frequencies of either control or DKO (CD45.2^+^) and wildtype competitor (CD45.1^+^) derived DCs (CD11c^+^CII^+^) in the spleen of BMT recipients. Shown are the frequencies within the indicated gates. (**c**) Frequencies of either control or DKO (CD45.2^+^) and wildtype competitor (CD45.1^+^) derived DCs in the spleen of mixed BMT recipients (n = 9–15) after 8 weeks. Indicated are the overall frequencies within the organ. (**d**) FACS plots indicating frequencies of either control or DKO (CD45.2^+^) and wildtype competitor (CD45.1^+^) derived cDC1 (CD8^+^CD11c^+^CII^+^) and cDC2 (CD11b^+^CD11c^+^CII^+^) subsets in the spleen of BMT recipients after 8 weeks. Shown are the frequencies within the indicated gates. (**e**) Frequencies of either control or DKO (CD45.2^+^) and wildtype competitor (CD45.1^+^) derived cDC1 (CD8^+^CD11c^+^CII^+^) (left) and cDC2 (CD11b^+^CD11c^+^CII^+^) (right) subsets in the spleen of BMT recipients (n = 9–15) after 8 weeks. Indicated are the overall frequencies within the organ. (**f**) FACS plots indicating frequencies of either control or DKO (CD45.2^+^) and wildtype competitor (CD45.1^+^) derived pDCs (CD11c^+^PDCA1^+^) in the spleen of BMT recipients after 8 weeks. Shown are the frequencies within the indicated gates. (**g**) Frequencies of either control or DKO (CD45.2^+^) and wildtype competitor (CD45.1^+^) derived pDCs (CD11c^+^PDCA1^+^) in the spleen of BMT recipients (n = 9–15) after 8 weeks. Indicated are the overall frequencies within the organ. (**h**) FACS plots indicating frequencies of either control or DKO (CD45.2^+^) and wildtype competitor (CD45.1^+^) derived cDC1 (XCR1^+^Sirpα^−^ CD11c^+^CII^+^) and cDC2 (XCR1^−^Sirpα^+^CD11c^+^CII^+^)subsets in the spleen of BMT recipients after 8 weeks. Shown are the frequencies within the indicated gates. (**i**) Frequencies of either control or DKO (CD45.2^+^) and wildtype competitor (CD45.1^+^) derived cDC1 (XCR1^+^Sirpα^−^ CD11c^+^CII^+^) (left) and cDC2 (XCR1^−^Sirpα^+^CD11c^+^CII^+^) (right) subsets in the spleen of BMT recipients (n = 9–15) after 8 weeks. Indicated are the overall frequencies within the organ. (**j**) Frequencies of either control or DKO (CD45.2^+^) and wildtype competitor (CD45.1^+^) derived DCs (CD11c^+^CII^+^) in the thymus of BMT recipients (n = 9–15) after 8 weeks. Indicated are the overall frequencies within the organ. (**k**) Frequencies of either control or DKO (CD45.2^+^) and wildtype competitor (CD45.1^+^) derived DCs (CD11c^+^CII^+^) in the BM of BMT recipients (n = 9–15) after 8 weeks. Indicated are the overall frequencies within the organ. (**l**) Frequencies of either control or DKO (CD45.2^+^) and wildtype competitor (CD45.1^+^) derived pDCs (CD11c^+^PDCA1^+^) in the BM of BMT recipients (n = 9–15) after 8 weeks. Indicated are the overall frequencies within the organ. (**m**) Frequencies of either control or DKO (CD45.2^+^) and wildtype competitor (CD45.1^+^) derived cDC1 (CD11c^+^PDCA1^−^CD8^+^CD11b^−^) (left) and cDC2 (CD11c^+^PDCA1^−^CD8^−^CD11b^+^) (right) subsets in the BM of BMT recipients (n = 9–15) after 8 weeks. Indicated are the overall frequencies within the organ. (**n**) FACS plots indicating frequencies of total CD11c^+^CII^+^ cDCs (left) and frequencies of wildtype competitor (CD45.1^+^) derived and either control or DKO (CD45.2^+^) derived cells within cDCs of the spleen from same animal, respectively, after 8 weeks. (**o**) Frequencies of wildtype competitor (CD45.1^+^) derived and either control or DKO (CD45.2^+^) derived CD11c^+^CII^+^ cDCs of the spleen from same animal, respectively, after 8 weeks (n = 9–15). Data represent mean and s.e.m. Two-tailed student’s t tests were used to assess statistical significance (*P < 0.05, **P < 0.01, *** < 0.001, *** < 0.001, ^n.s.^ *P* > 0.05).

The original Article has been corrected.

