# Supplementary material for: Class I PI3K regulatory subunits control differentiation of dendritic cell subsets and regulate Flt3L mediated signal transduction
Source: Sci Rep. 2022 Jul 19;12:12311. doi: 10.1038/s41598-022-16548-x (PMC9296662; doi:10.1038/s41598-022-16548-x)
Supplement: Supplementary file 1 — Supplementary Information 1. [file 41598_2022_16548_MOESM1_ESM.pdf]

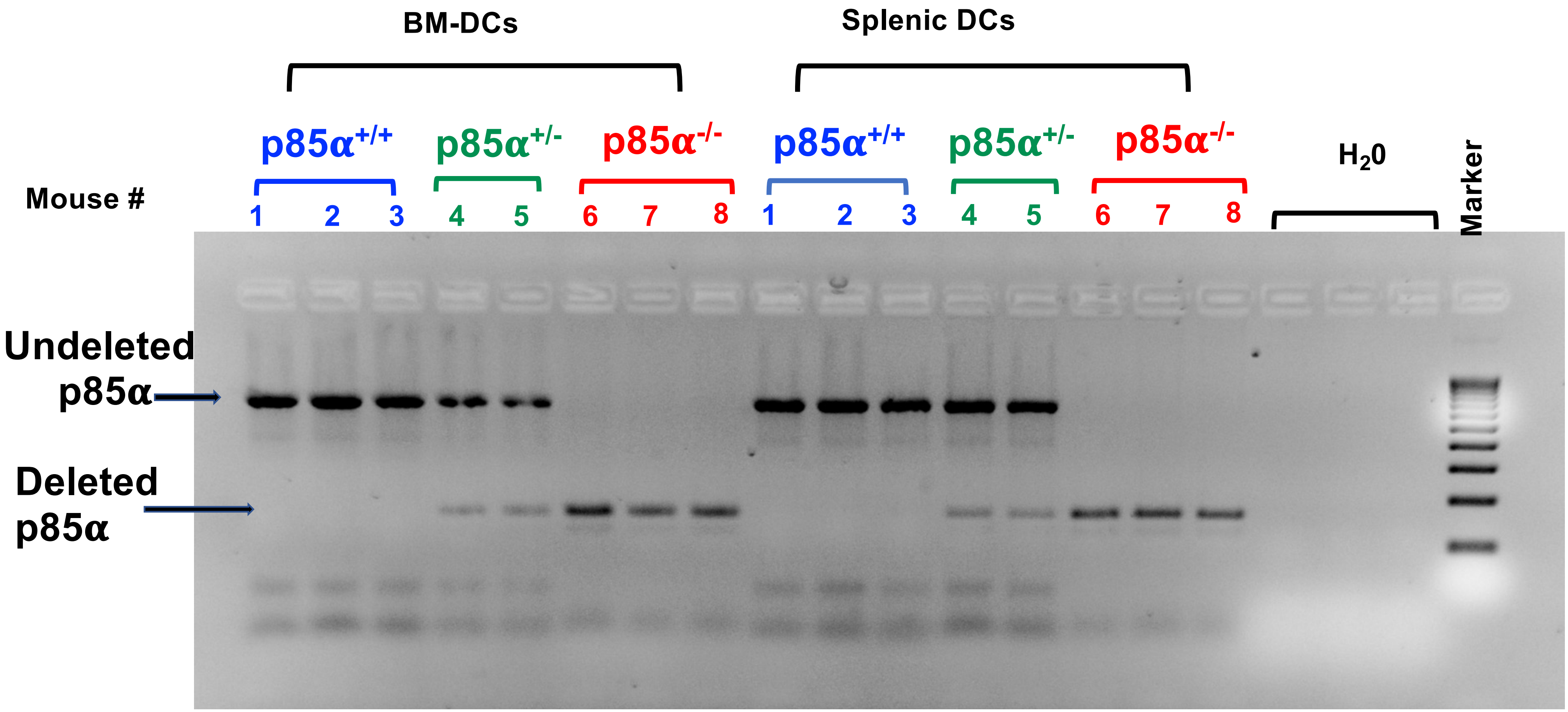

Original gel image of Supplemental Figure 2b.

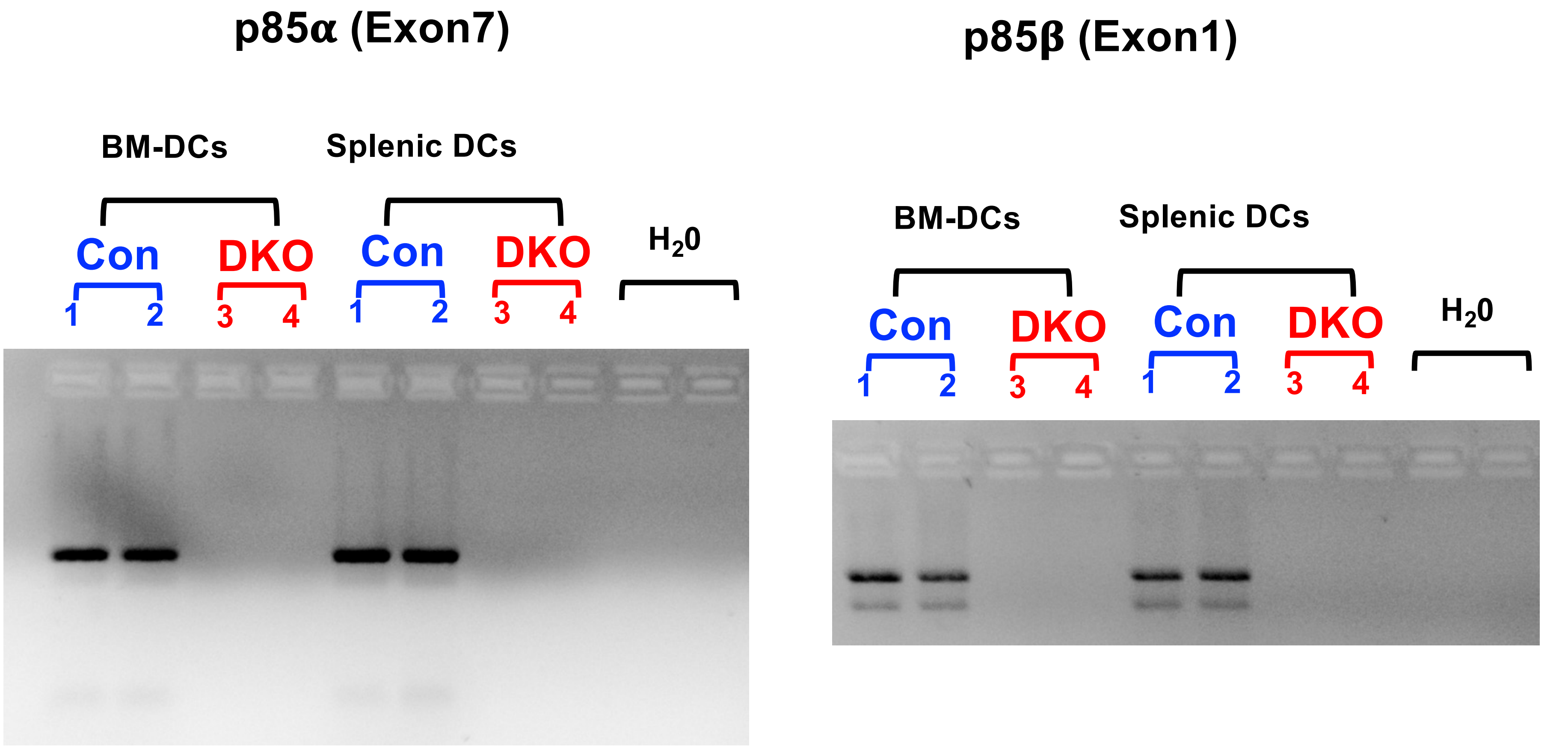

Original gel images of Supplemental Figure 2c.

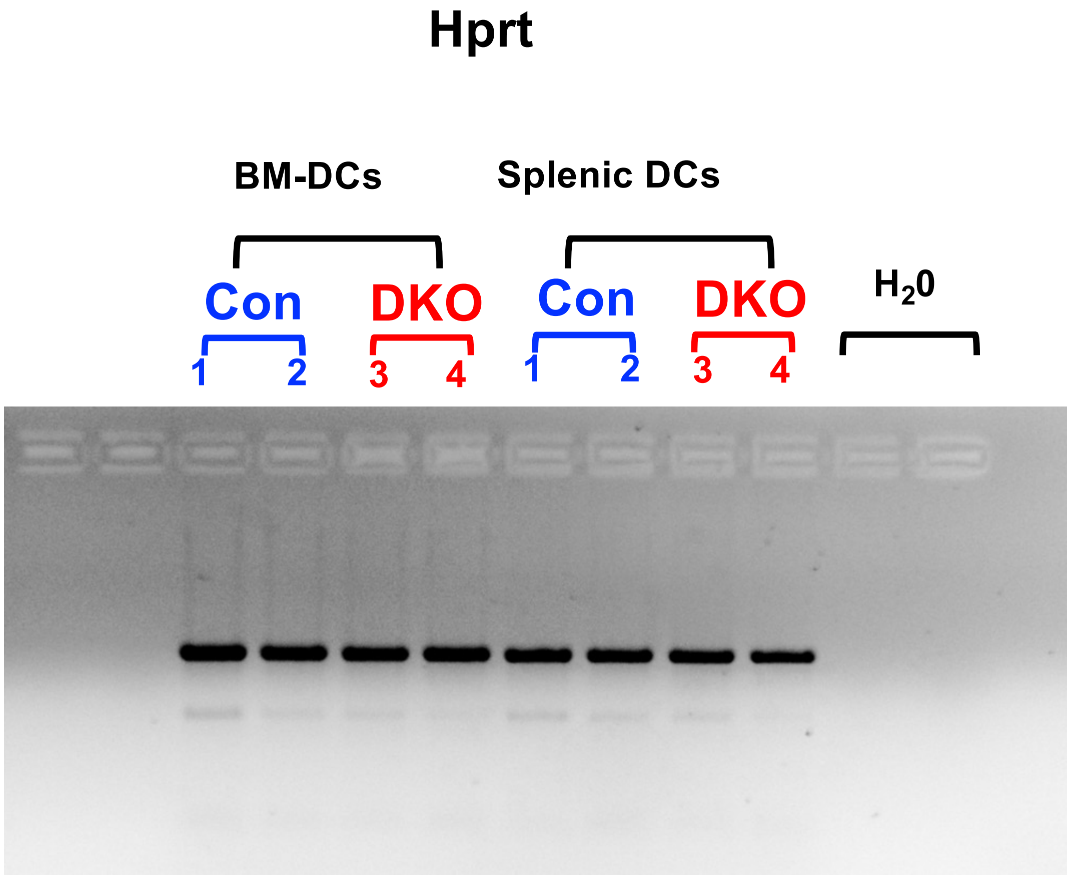

Original gel images of Supplemental Figure 2c.
