# Supplementary material for: Class I PI3K regulatory subunits control differentiation of dendritic cell subsets and regulate Flt3L mediated signal transduction
Source: Sci Rep. 2022 Jul 19;12:12311. doi: 10.1038/s41598-022-16548-x (PMC9296662; doi:10.1038/s41598-022-16548-x)
Supplement: Supplementary file 3 — Supplementary Information 3. [file 41598_2022_16548_MOESM3_ESM.pdf]

## Supplementary Figures Legend.

### Supplementary Figure 1.

**a**. FACS plots indicating frequencies of granulocytes ($CD11b^+Ly6G^+$) and monocytes/ macrophages ($CD11b^+Ly6G^-$) in the BM of 4-week old mice.

**b**. Frequencies of granulocytes ($CD11b^+Ly6G^+$) and monocytes/ macrophages ($CD11b^+Ly6G^-$) in the BM of 4-week old mice (n= 5-9).

**c**. FACS plots indicating frequencies of Erythrocytes ($Ter119^+$) in the BM of 4-week old mice.

**d**. Frequencies of Erythrocytes ($Ter119^+$) in the BM of 4-week old mice (n= 5-9).

**e**. FACS plots indicating frequencies of Megakaryocytes ($CD41^+$) in the BM of 4-week old mice.

**f**. Frequencies of Megakaryocytes ($CD41^+$) in the BM of 4-week old mice (n= 5-9).

**g**. FACS plots indicating frequencies of B cells ($CD19^+$) in the BM of 4-week old mice.

**h**. Frequencies of B cells ($CD19^+$) in the BM of 4-week old mice (n= 5-9).

**i**. Surface expression levels of CD80, CD86 and CD40 from the BM of DKO and control mice cultured *in-vitro* in the presence of GM-CSF for 7 days (n=11). Cells were pre-gated on $CD11c^+CII^{high}$ cells. Shown are Geomean fluorescence Intensities (GMFI) of surface markers.

**j**. Surface expression levels of CD80, CD86 and CD40 from the BM of DKO and control mice cultured *in-vitro* in the presence of GM-CSF for 7 days and stimulated with TNF$\alpha$ for 24 hours (n=11). Cells were pre-gated on $CD11c^+CII^{high}$ cells. Shown are Geomean fluorescence Intensities (GMFI) of surface markers.

**k**. Surface expression levels of MHC-Class I, MHC-Class II, CD80, CD86 and CD40 from the BM of DKO and control mice cultured *in-vitro* in the presence of Flt3L for 14 days (n=11). Cells were pre-gated on $CD11c^+CD11b^+$, $CD11c^+CD11b^+CD45RA^+CLEC9A^-$, $CD11c^+CD11b^+CD45RA^-CLEC9A^+$cells. Shown are Geomean fluorescence Intensities (GMFI) of surface markers.

### Supplementary Figure 2.

**a.** Histograms indicating EGFP fluorescence in total DCs, cDC1, cDC2 and pDC subsets of the spleen from Control (**Top**) and $Rosa^{GFP}$ $Vav^{cre}$ (**bottom**) mice. Data are representative of 5 independent experiments. Shown are the frequencies of cells within the $GFP^+$ gate.

**b.** Representative image of agarose gel indicating the profiles of PCR products that were amplified using genomic DNA obtained from MACS sorted $CD11c^+$ DCs of BM and spleen from control ($p85\alpha^{+/+}p85\beta^{+/+}$), heterozygous ($p85\alpha^{F/+}p85\beta^{+/-}Vav^{Cre}$) and DKO ($p85\alpha^{F/F}p85\beta^{-/-}$ $Vav^{Cre}$) mice. # indicates genomic DNA samples from independent animals.

**c.** Representative images of agarose gel indicating the RT-PCR products that were amplified using cDNA obtained from MACS sorted CD11c$^{+}$ DCs of BM and spleen of control (p85α$^{+/+}$p85β$^{+/+}$) and DKO (p85α$^{F/F}$p85β$^{-/-}$ Vav$^{Cre}$) mice. Each lane indicates cDNA samples from a pool of DCs from two animals .

**Supplementary Figure 3.**

**a.** Frequencies (n=8-11) of cDC, cDC1, cDC2 and pDC subsets in the spleen of 6-8 weeks old control and p85α$^{F/F}$Vav$^{Cre}$ mice.

**b.** Frequencies (n=8-11) of cDC, cDC1, cDC2 and pDC subsets in the spleen of 6-8 weeks old control and p85β$^{-/-}$ mice.

**c.** Frequencies (n=8-11) of cDC, pDC, cDC1 and cDC2 subsets in the BM of 6-8 weeks old control and p85α$^{F/F}$Vav$^{Cre}$ mice.

**d.** Frequencies (n=8-11) of cDC, pDC, cDC1 and cDC2 subsets in the BM of 6-8 weeks old control and p85β$^{-/-}$ mice.
Data represent mean and s.e.m. Two-tailed student's t tests were used to assess statistical significance (*P < 0.05, **P<0.01, $^{n.s.}$ P > 0.05).

**Supplementary Figure 4.**

**a.** Frequencies (n=9) of total cDCs in the spleen of 6-8 weeks old control and Pi3kr1$^{F/+}$Vav $^{Cre/+}$ mice.

**b.** Frequencies (n=8) of cDC1 in the spleen of 6-8 weeks old control and Pi3kr1$^{F/+}$Vav $^{Cre/+}$ mice.

**c.** Frequencies (n=9) of cDC2 in the spleen of 6-8 weeks old control and Pi3kr1$^{F/+}$Vav $^{Cre/+}$ mice.

**d.** Frequencies (n=11) of pDCs in the spleen of 6-8 weeks old control and Pi3kr1$^{F/+}$Vav $^{Cre/+}$ mice.

Data represent mean and s.e.m. Two-tailed student's t tests were used to assess statistical significance (*P < 0.05, **P<0.01, $^{n.s.}$ P > 0.05).

**Supplementary Figure 5.**

**a.** Frequencies (n=11-21) of total cDCs in the BM of 6-8 weeks old control and Pi3kr1$^{F/+}$Vav $^{Cre/+}$ mice.

**b.** Frequencies (n=16-17) of pDCs in the BM of 6-8 weeks old control and Pi3kr1$^{F/+}$Vav $^{Cre/+}$ mice.

**c.** Frequencies (n=12-23) of PDCA1$^{-}$CD11c$^{+}$ DCs in the BM of 6-8 weeks old control and Pi3kr1$^{F/+}$Vav $^{Cre/+}$ mice.

**d.** Frequencies (n=10-12) of PDCA1$^-$CD11c$^+$CD8$^+$ DCs in the BM of 6-8 weeks old control and Pi3kr1$^{F/+}$Vav $^{Cre/+}$ mice.

**e.** Frequencies (n=11-29) of PDCA1$^-$CD11c$^+$CD11b$^+$ DCs in the BM of 6-8 weeks old control and Pi3kr1$^{F/+}$Vav $^{Cre/+}$ mice.

**f.** Frequencies (n=6-16) of PDCA1$^-$CD11c$^+$CD11b$^-$CD8$^-$ DCs in the BM of 6-8 weeks old control and Pi3kr1$^{F/+}$Vav $^{Cre/+}$ mice.

Data represent mean and s.e.m. Two-tailed student's t tests were used to assess statistical significance (*$P < 0.05$, **$P<0.01$, $^{n.s.}$ $P > 0.05$).

**Supplementary Figure 6. Pre-gating scheme of flow cytometric analysis of BM, Spleen and thymus.**
